# Engagement with life and psychological well-being in late adulthood: Findings from community-based programs in Portugal

**DOI:** 10.1371/journal.pone.0286115

**Published:** 2023-05-19

**Authors:** Alice Bastos, Joana Monteiro, Raquel Barbosa, Helena Pimentel, Sofia Silva, Carla Faria

**Affiliations:** 1 School of Education, Polytechnic Institute of Viana do Castelo, Viana do Castelo, Portugal; 2 CINTESIS–Center for Health Technology and Services Research, Universidade do Porto, Porto, Portugal; 3 Faculty of Psychology and Education Sciences at University of Porto, Center for Psychology at University of Porto, Porto, Portugal; 4 Health School, Polytechnic Institute of Bragança, Bragança, Portugal; 5 Polytechnic Institute of Bragança, Health Sciences Research Unit: Nursing (UICISA: E), Bragança, Portugal; 6 School of Education, Polytechnic Institute of Coimbra, Coimbra, Portugal; 7 Polytechnic Institute of Coimbra, Centre for 20th Century Interdisciplinary Studies (CEIS20), University of Coimbra, Research Group in Social and Human Sciences, Human Potential Development Center (CDPH), Coimbra, Portugal; Taipei Medical University, TAIWAN

## Abstract

**Objectives:**

Human aging is a multidirectional, multidimensional, and multicausal process that reflects biological, psychological, and sociocultural influences, which act in distinct combinations throughout the life-span. Proactivity towards avoiding the usual aging process is needed. This study analyses the long-term effects of participation in Community-Based Programs on psychological well-being.

**Method:**

A sample of 150 community-dwelling participants enrolled in Community-Based Programs, aged 55 to 84 years and living in three Portuguese localities were matched by age (55–64, 65–74, 75–84 years), gender, and locality with a comparison group of non-participants. We administered a multidimensional gerontological protocol which included socio-demographic information, measures of health/disease, functional ability, social network, cognitive performance and psychological well-being. Hierarchical regression models were used to test the effects of Community-Based Programs on psychological well-being adjusting for remaining variables.

**Results:**

Overall, psychological well-being is positively associated with household income and satisfaction with health. Nevertheless, in participants, psychological well-being builds predominantly upon social network and is not associated with a moderate inability or cognitive deficits, contrasting with psychological well-being in non-participants. After adjusting for background variables, psychological well-being was positively associated with health satisfaction and social network and negatively related to moderate inability. Further, a significant interaction of participation in Community-Based Programs with age, points out higher levels of psychological well-being in participants contrasting with a downward trend in non-participants. After stratification by age, psychological well-being increases with time attending Community-Based Programs in the oldest (75–84 years) contrasting with the remainder.

**Conclusions:**

Participation in Community-Based Programs may improve the negative effects of the aging process on psychological well-being. This positive effect as age increases may be linked to a reinforcement of social network, valued more by participants in Community-Based Programs. Furthermore, the programs may act as a healing/maintenance strategy in persons with moderate inability and/or cognitive deficits.

## Introduction

Human aging is one of the major societal challenges in the 21^st^ century. Aging is a multidirectional, multidimensional, and multicausal process that reflects biological, psychological, and sociocultural influences, which act in distinct combinations throughout the life-span [1,2]. Each individual has an inherent potential to change and to develop reserve capacity. However, personal choices have different outcomes depending on life circumstances and the moment each of us is going through in life [3].

In the last two decades of the 21^st^ century, there has been a tendency in Gerontology to think about aging in a *positive* way. Fernández-Ballesteros [4] observed that the term “healthy” (*n* = 2.523 papers) prevailed over “successful” (*n* = 1.447 papers). Also, a positive perspective on aging (“active aging” or “healthy aging”) is present in the World Health Organization policy framework [5–7].

Theoretically, several successful aging theories and models have been guiding research, as with Life-Span Developmental Psychology proposed by Baltes and cols [8,9]. At the same time, this positive perspective is embodied in Rowe and Kahn’s [10–12] successful aging model, and in the Preventive and Corrective Proactivity model by Kahana and Kahana [13–15]. According to Caprara and Mendoza-Ruvalcaba [2, p. 512], “the notion of successful aging, in particular, has been crucial to promote a new paradigm that acknowledges the value of aging well and counteracts a vision of loss and deficit (…)”. The same authors state that aging well should be promoted so that individuals make the best use of their potential. At the same time, it should be noted that life contexts play a central role in aging. The time and space in which human development occurs determine the nature and extent of individual opportunities and limitations. Caprara and Mendoza-Ruvalcaba [2, p. 513] also stress that scholars like Ryff, Rowe and Khan, and Baltes and Baltes proposed new perspectives on aging that “led to the need for policies and programs designed to prolong and sustain old people’s positive engagement in their communities”.

Thus, a developmental view of successful aging is distinct in that it accounts for the multiple influences (normative and non-normative) associated with age and cohort on human development/aging.

In the ‘80s, Carol Ryff [16] proposed a developmental approach to successful aging. Globally, the author sustained that different life periods have different agendas and that research should consider this. During that decade, Ryff [17,18] developed a model to explain psychological well-being (PWB) throughout the life cycle and created a set of scales to measure PWB, questioning dominant conceptions until then, which focused on happiness, positive affect, and satisfaction with life. Conversely, in Ryff’s perspective, PWB is understood as attaining individual potential, feelings of purpose and direction in life. Her vision became known in the literature as the eudaimonic perspective on well-being, as opposed to the previous hedonic view [19,20]. Importantly, Ryff highlights the possibility of growth and development during old age, in line with the Life-span Developmental Psychology endorsed by Baltes [21]. In this way, new opportunities emerged to approach successful aging as a human co-construction dependent on the double person-context interaction [22,23].

According to Ryff’s model [17,18,24,25], PWB is multidimensional, encompassing six dimensions subsequently explained. *Self-acceptance* means cultivating positive attitudes towards oneself and one’s past life, acknowledging and accepting personal characteristics. In its turn, *positive relations with others* refers to satisfying social relationships, with affection and trust, as well as intimacy and empathy. Thirdly, *autonomy* indicates independence and self-determination; the ability to resist social pressures and regulate behavior from within. Another dimension is *environmental mastery*, which entails a sense of competence to manage life situations, creating and dealing with contexts according to personal needs and values. Moreover, *purpose in life* means having future goals and plans, as well as a sense of directedness and feeling that present and past life have meaning. Finally, *personal growth*, linked with openness to experience, corresponds to a sense of fulfilling individual potential and seeing oneself as roving throughout time, changing and developing continually.

Research about PWB using Ryff’s scales [25,26]. shows distinct tendencies between young, middle-aged, and older adults. It was observed that middle-aged adults revealed higher scores in some dimensions (for example, purpose in life, personal growth), while older adults displayed better results than young adults in environmental mastery and autonomy but lower scores than middle-aged adults in personal growth and purpose in life. Similar tendencies were observed in the Portuguese population for personal growth and purpose in life [23,27]. These results defy the more pessimistic views about the aging process and, at the same time, warn for possible specific old age challenges.

It should be noted that in the MIDUS longitudinal study, Ryff, Radler, and Friedman [28] observed different PWB profiles in adults, stating that participants with continually high PWB exhibited better health than those who displayed continually reduced PWB. The authors highlight the importance of intervention to promote population’s well-being.

Regarding the associations between engagement with life and PWB, a recent longitudinal study with Ryff’s PWB scales showed that well-being reduces slightly but consistently between around the mid-50s and the mid-70s years of age [29]. Simultaneously, it evinced that individuals with higher social participation reported higher initial PWB levels, and a smaller decline in PWB throughout time, demonstrating that social participation has a protective effect on well-being.

As mentioned by Sharifian and Grun [29], research using Ryff’s PWB scales to evaluate community-based programs aiming to promote successful aging is not common. Nevertheless, Friedman et al. [30] tested the effects of a community-based program in a sample of 103 participants aged 60 years or older. This eight-week program aims to teach participants to identify and savor positive experiences in several eudaimonic well-being domains. Results showed that participants reported a significant increase in PWB, satisfaction with life, and social well-being, along with lower depression levels and fewer physical symptoms, and sleep complaints. These gains were particularly robust in individuals with lower PWB levels before the program. This study suggests the viability of group interventions to improve well-being in older adults. In turn, Weiss, Westerhof, and Bohlmeijer [31] performed a meta-analysis about individual and small-group intervention effects on PWB, based on eudaimonic well-being as proposed by Ryff. This meta-analysis used only studies of behavioral interventions with a comparison group (RCT) that used Ryff’s PWB scales or the Mental Health Continuum–Brief Form. Results showed that interventions were more effective in clinical groups and when performed individually. According to this meta-analysis, it seems possible to improve PWB with behavioral interventions, even though effects were more substancial in studies with a higher risk of bias.

According to Caprara and Mendoza-Ruvalcaba [2], promoting successful aging requires making people acknowledge the harmful effects of unhealthy habits and adopt new habits (physical activity). Optimal cognitive functioning can be achieved through life learning and engagement in activities that enhance memory, judgment, and problem-solving activities. Concerning self-system, researchers have identified a great variety of self-constructs, namely “life satisfaction”, self-regulation, self-efficacy [and “happiness”, psychological well-being”] as core personal determinants factors of SA. Also, findings suggest that nurturing positive emotions (e.g., joy and pride) may promote well-being and balance negative emotions (e.g., anxiety and depression). Empirical evidence shows a positive link between social activity and participation and cognitive functioning, but the protective effects of social engagement tend to reduce over time.

In summary, Ryff’s PWB scales have been used in research and intervention (individual and small groups). Nonetheless, community-based intervention studies have been absent from research. Therefore, it is time to investigate these aspects. On the other hand, it is also important to investigate possible shortcomings, so future work in this field could address other objectives and community dwellers. Community-intervention programs have in common to enhance citizens’ participation in diverse activities, promoting social relations whenever job and family relations become less frequent, after retirement, and when children leave the parental home. These programs are in line with the WHO’s active and healthy aging policy frameworks [5–7]. Even though they are not mainly designed to promote PWB as other individual designed interventions, we may expect these programs to boost PWB. One of our objectives is to characterize the population they attract and also to explore the possible benefits they generate for PWB in the long run. In this study we propose to test several hypotheses: H1: Participation in CBP improves PWB, namely personal growth and purpose in life, irrespective of the socio-demographic profile, health, and social network measures; H2: Participation in CBP improves PWB, depending on the socio-demographic profile, namely counteracting deleterious effects of aging, low education or low income. To corroborate our findings, we tested the possible effects of the duration of the intervention (years since the beginning of involvement in activities) on PWB.

## Methods

The present study is part of a multicentre and multimethod research project developed in three territories from Northern and Centre Portugal (*AgeNortC–Aging*, *social participation*, *and early dependency detection*: *empowering for the 4*^*th*^ *Age*). This paper refers to a quantitative cross-sectional study focused on Community-Based Programs (CBP) implemented by municipal councils as active/successful aging promotion actions. In this context, participation in such programs is considered to be an expression of engagement with life in late adulthood. These programs provide opportunities to engage in group activities of diverse nature, such as physical (e.g. exercise classes, water aerobics), sociocultural (e.g. social dance, workshops), and lifelong learning/education (e.g. reading and writing, medicinal and aromatic plants garden). These activities are implemented by municipalities to operationalize governmental measures aimed at improving population health, quality of life and well-being. Such governmental measures are in line with the WHO’s active and healthy aging policy frameworks [5–7], and stem from the Portuguese National Strategy for Active and Healthy Aging.

### Participants and data collection procedures

This cross-sectional study included 152 community-dwelling participants enrolled in CBP aged 55 to 84 years and living in three localities of Portugal–one in the North Coast (n = 52), one in the Interior North (n = 50) and one in the Centre region (n = 50), as displayed in S1 Table. Participants were recruited through direct contact in facilities where CBP’s activities took place or via parish councils/associations. Each participant from the CBP was matched by age (55–64, 65–74, 75–84 years) and gender with a family member, someone from the neighborhood network, or via parish councils/associations within the same territory/community, to recruit a comparison group of participants that did not attend CBP.

Based on previously published results using Ryff’s PWB scales [32], an overall sample size of approximately 150 participants in CBP and an equal number in a comparison group would enable a unit difference to be detected in PWB dimensions with 95% confidence and power greater than 80%.

The study was conducted according to the Declaration of Helsinki. All participants received information about study goals and data collection procedures. They were also informed regarding participation conditions, including confidentiality, voluntary collaboration and the possibility of ceasing their involvement in the study at any point in time without need for justification. Subsequently, all participants gave their written informed consent. Data collection was carried out by researchers and research fellows (n = 9) with the collaboration of undergraduate and master’s students in Social Gerontology (n = 9) from the three higher education institutions involved in this study. The entire team underwent previous training. A multidimensional gerontological evaluation protocol was designed and administered at previously agreed sites with adequate privacy conditions (higher education institutions and community infrastructures), between March and April 2018.

### Measures

The multidimensional gerontological evaluation protocol included a questionnaire developed by the research team with two sections: (1) sociodemographic characteristics—21 close-ended questions and (2) aspects of participation in CBP—six close-ended questions. Concerning participation in CBP, this questionnaire asks about: (1) activity type (physical, sociocultural, lifelong leraning) and number; (2) frequency of involvement in activities (once a month, once a week, twice a week, three or more times a week); (3) duration (number of years attending activities); (4) motives for participation (stay healthy/medical advice, keep myself busy, meet new people, enjoy myself, keep fit).

This information was complemented by measures of health status, disability in activities of daily living, cognitive performance, social network and PWB.

### Input measures

Input measures encompassed the following sociodemographic aspects: age, gender, marital status, education level, and monthly household income. This information was collected with the questionnaire mentioned earlier.

Additionally, health status was assessed with one item from the Portuguese version of the World Health Organization Quality of Life-Bref Instrument (WHOQOL-BREF) [33,34]. The used item asks about an individual´s overall satisfaction with personal health–“How satisfied are you with your health?”–, with response scores ranging from 1 (very dissatisfied) to 5 (very satisfied). Participants whose answers were 1 or 2 were considered dissatisfied with their health.

The Instrumental Activities of Daily Living Scale (IADL) [35], Portuguese version by Sequeira [36], allowed the assessment of functional ability. The IADL consists of eight items comprising eight instrumental activities of daily living: housekeeping, laundry, food preparation, shopping, telephone use, transportation, ability to handle finances, and responsibility for personal medication. For each item, there are three to five response options from independence to different dependency levels. The scores range from 8 to 30, with higher scores corresponding to more dependency on instrumental activities of daily living. According to established cut-off points, a score of 8 points corresponds to independence, a score from 9 to 20 to moderate dependency, and a score >20 to severe dependency. Since a score of 9 indicates some degree of dependency, in this study, needing help or assistance in at least one instrumental activity of daily living was considered as a functional disability. The Portuguese IADL shows good internal consistency (*α* = .92), with principal components analysis showing a one-factor solution that explains 65% of the total variance [36].

Cognitive performance was measured using the Mini-Mental State Examination (MMSE) [37] Portuguese version [38]. The MMSE focuses on global cognitive functioning and is widely used for the purpose of cognitive impairment screening with older adults. It is composed of 30 items that assess six cognitive domains: orientation, retention, attention and calculation, recall, language, and constructive ability. Items are scored with 1 point if correct and 0 points if incorrect, with global scores ranging from 0 to 30 points. Higher scores represent better cognitive performance. MMSE results vary according to sociodemographic variables such as age and education. In 2009, Morgado et al. [39] published a new psychometric study of this instrument in the Portuguese context, in which years of formal education were the most relevant factor influencing MMSE performance. The authors then updated the instruments’ cut-off points considering three education levels: (1) 0 through 2 years of school– 22 points; (2) 3 through 6 years of school– 24 points; (3) 7 or more years of school– 27 points. Results under the mentioned limits signal the risk of cognitive impairment, albeit this does not distinguish healthy and cognitively impaired individuals since this is a screening test. Morgado et al. [39] found a moderate internal consistency (*α* = .46) that might be due to the fact that the MMSE measures different cognitive domains.

Social network and integration were measured with the Portuguese version of the Lubben Social Network Scale (LSNS-6) [40,41]. The used version comprises six items and has two subscales–family and friends–, with three items each. The individual is asked the same three questions regarding family network and friends/neighbours network. These questions focus on the network dimension, perception of support availability, and confidants. The respondent indicates the number of persons in his/her social network for each of the mentioned aspects, using one of six options (from 0 to 9 or more). Responses are scored from 0 to 5 points. Hence, scores range from 0 to 30 for the global scale, and from 0 to 15 for each subscale. The original and the Portuguese versions indicate a score of 12 points as the cut-off point below which an individual is at risk for social isolation. The Portuguese version of the LSNS-6 [41] displayed adequate internal consistency for the global scale (*α* = .80), as well as for the family (*α* = .76) and friends (*α* = .73) subscales.

### Outcome measures

The main study outcome was psychological well-being assessed through The Psychological Well-Being Scales developed by Ryff (PWBS) [18,26] and adapted to the Portuguese population by Novo, Silva, and Peralta [27]. The used version encompasses 18 items, with Likert response options from 1 (completely disagree) to 6 (completely agree). The PWBS measure psychological well-being according to Ryff’s eudaimonic perspective, with six subscales corresponding to the six well-being dimensions: self-acceptance, positive relations with others, autonomy, environmental mastery, purpose in life, and personal growth. Each dimension encompasses three items, with scores ranging from 3 to 18 points. The overall score ranges from 18 to 108 points. Higher scores correspond to higher well-being levels. The psychometric studies of the Portuguese version [27] were based on an 84-item scale. They showed good internal consistency regarding the global scale (*α* = .93) and the subscales (*α* values between .74 and .86), as well as good temporal stability. The present study used the overall, purpose in life and personal growth scales.

### Data analysis

Following the description of activities offered by CBP and participation in terms of frequency, motivations, and duration, we compared individual characteristics of participants and non-participants in CBP using standard tests (chi-square for qualitative and t-test for quantitative data). Characteristics associated with PWB in both groups were based on Pearson correlation coefficients. A hierarchical linear regression model was used to test whether involvement in CBP bears any relation with PWB controlling for effects of other background variables; the first block included socio-demographic variables (Model 1), the second block included health and social network measures (Model 2), and the third block included interactions of participation with all socio-demographic variables (Model 3). Whenever an interaction was significant, we performed a sub-group analysis, investigating diverse effects on overall PWB and its dimensions. A *p*-value of .05 was considered the limit for wrongly rejecting the null hypothesis.

## Results

As shown in Table 1, physical activities were more often offered and participated (78.3%) followed by sociocultural and lifelong learning activities in a similar proportion (26%). Overall, 112 (73.7%) individuals participated in just one type of activity, mainly physical activity (59.9%), 34 (22.4%) in two types of activities, and 6 (3.9%) in all three types. Only 33 (21.7%) participants were not engaged in physical activities. Looking at single activities, 96 (63.2%) participated in two or more and 87 (57.2%) at least twice a week. About 97% of participants stated attending activities assiduously. More than two-thirds of participants stated as participation motivation keeping themselves healthy, and just over 40% stated being occupied or meeting new people. Participation ranges from less than one year (19.1%) to 10 or more years (17.1%), but most have been attending activities for at least three years (61.8%).

**Table 1:** Participation in activities offered in community-based programs and psychological well-being (*N* = 152).

	*N*	%	PWB *M* (*SD*)
Physical activities^a^	119	78.3	80.9 (10.4)
Exercise classes	102	67.1	
Swimming and water aerobics classes	81	53.3	
Walking clubs	8	5.3	
Sociocultural activities^a^	40	26.3	80.8 (10.9)
Social dance	23	15.1	
Workshops	19	12.5	
Theatre/Cinema	8	5.3	
Lifelong learning activities^a^	39	25.7	81.3 (12.6)
Computing	19	12.5	
Greencare activities	14	9.2	
Reading & Writing (library clubs)	9	5.9	
Number of activities			
One	56	36.8	78.6 (10.6)
Two	71	46.7	81.3 (10.4)
Three	20	13.2	80.2 (11.7)
Four or more	5	3.3	87.4 (12.5)
Frequency of participation			
Once a month	19	12.5	84.2 (11.6)
Once a week	46	30.3	79.1 (10.9)
Twice a week	66	43.4	80.9 (10.0)
Three or more times a week	21	13.8	78.2 (11.6)
Motive for participation^a^			
Stay healthy/medical advice	102	67.1	79.9 (11.1)
Keep myself busy	68	44.7	80.9 (10.8)
Meet new people	62	40.8	82.6 (9.8)
Enjoy myself	49	32.2	82.6 (8.7)
Keep fit	47	30.9	81.8 (9.8)
Duration of participation, years^b^			
≤1	38	25.0	78.2 (12.2)
2–4	61	40.1	79.8 (10.0)
≥5	53	34.9	82.7 (10.3)

^a^ Activities and motives are multiple (% do not add 100).

^b^ Duration of participation: Test for linear trend–*F*(1,150) = 4.18, *p* = .043.

The mean age of participants is 71.4 years (*SD* = 5.7), most are women (75.0%),about two-thirds (64.5%) are married, and 25.7% are widowed (Table 2). Most participants are retired (90.1%) and education level is low, about 70.0% have at most four years of full-time education and only 11.8% have ten years or more. Average household income does not reach two minimum wages.

**Table 2:** Characteristics of CBP participants and non-participants: % or Mean (SD), and [Range].

	All (N = 304)	PG (n = 152)	N-PG (n = 152)	Test of differences
Age, years	71.5 (5.7)	71.4 (5.4)	71.6 (6.1)	
Gender: Female	75.0	75.0	75.0	
Marital status: Married	67.1	64.5	69.7	1.0
Education level, years	5.3 (3.5)	5.2 (3.5)	5.3 (3.5)	0.2
		[0–22]	[0–17]	
Monthly household income (€)	1.7 (1.2)	1.6 (1.0)	1.9 (1.3)	2.1*
Self-reported chronic conditions ≥ 2	10.5	9.2	11.8	0.6
No of doctor visits past year	5.7 (6.3)	6.0 (7.1)	5.3 (5.5)	1.0
No of doctor visits >6	25.3	25.0	25.7	0.0
Satisfaction with health	3.5 (0.9)	3.5 (0.9)	3.4 (0.9)	0.8
		[0–5]	[0–5]	
Dissatisfied	14.1	12.5	15.8	0.7
Functional ability (IADL)	9.5 (2.2)	9.2 (1.8)	9.7 (2.5)	2.0*
		[8–16]	[8–20]	
Dependency in IADL	47.4	46.1	48.7	0.2
Cognitive function (MMSE)	26.8 (2.7)	26.8 (2.6)	26.8 (2.8)	0.0
		[15–30]	[17–30]	
Cognitive deficit	13.5	13.2	13.8	0.0
Social Network (LSNS-6)	17.7 (5.6)	17.7 (5.6)	17.7 (5.6)	0.0
		[3–30]	[6–30]	
Risk of social isolation	15.1	13.8	16.4	0.4
Psychological well-being (PWBS)	80.0 (10.6)	80.4 (10.7)	79.6 (10.4)	0.6
		[46–104]	[50–106]	
Personal growth	13.9 (2.4)	14.0 (2.4)	13.7 (2.4)	1.2
		[7–18]	[6–18]	
Purpose in life	13.0 (3.3)	13.3 (3.2)	12.7 (3.3)	1.5
		[3–18]	[3–18]	

PG–Participants group; N-PG–Non-participants group; test: Chi-square for qualitative data and t-test for quantitative data; monthly household income in units of National minimum wage.

* *p* < .05.

Half of the participants stated not having chronic conditions and musculoskeletal disorders, diabetes, and heart diseases were the most frequently reported; only 9.2% reported two or more chronic conditions. Most participants (53.9%) attended routine appointments at the health centre/hospital twice to five times in the past year. Only 12.5% stated not being satisfied with their health status in the past two weeks, although approximately 46% reported needing assistance in some routine daily activity. Most of them (30%) need assistance in at most two activities, and none have a severe disability. About 13% of participants scored less than the expected cut-off in cognitive function (MMSE) for their level of education, and only two scored less than 20. About 14% may be considered at risk of social isolation, though only six (3.9%) are simultaneously isolated from family and friends. As shown in Table 2, the characteristics of participants and non-participants are not significantly different, except for household income and mean IADL; CBP participants have lower income levels and are less dependent on instrumental activities of daily living than non-participants. PWB, personal growth, and purpose in life are slightly higher in participants than in non-participants, though differences do not reach statistical significance. Activities outside CBP are frequently and equally performed by participants and non-participants in CBP. Nevertheless, participants in CBP are more often members of associations (see S2 Table).

Table 3 describes characteristics associated with PWB, personal growth, and purpose in life in participants and non-participants in CBP. Higher PWB is associated with education, income, and satisfaction with health in both groups; age, restrictions on instrumental activities of daily living, and cognitive deficits are associated with lower PWB only in non-participants, and social network is associated with higher PWB only in participants. For this overall PWB pattern contribute both personal growth and purpose in life.

**Table 3:** Correlations of background variables with psychological well-being in participants and non-participants of CBP.

	PWB			Personal growth			Purpose in Life		
	All	PG	N-PG	All	PG	N-PG	All	PG	N-PG
Age (years)	-.07	.10	-.21**	-.19***	-.12	-.25**	-.04	.10	-.17*
Women^a^	-.09	-.06	-.12	-.05	-.02	-.08	-.09	-.06	-.12
Married^a^	.08	.03	.13	.01	-.03	.05	.13*	.07	.21**
Education (years)	.19**	.13	.24**	.25***	.21**	.29***	.18**	.14	.22**
Log income	.25***	.22**	.30***	.23***	.24**	.24**	.24***	.18*	.31***
Satisf. w/ health	.26***	.23**	.28***	.22***	.23**	.21**	.20***	.21**	.19*
Doctors’ visits > 6^a^	-.00	.09	-.10	-.03	.03	-.09	.01	.02	.01
Dependency in IADL^a^	-.21***	-.11	-.31***	-.15**	-.11	-.19*	-.15*	-.12	-.17*
Cognitive deficit^a^	-.15*	-.09	-.21*	-.06	-.12	-.01	-.14*	-.14	-.14
Social network	.24***	.35***	.13	.21***	.24**	.18*	.20**	.19*	.21**

PWB–Psychological well-being; PG–Participants group, N-PG–Non-participants group, Satisf. w/ h–Satisfaction with health, IADL–Instrumental activities of daily living.

^a^ category coded as 1.

* *p* < .05

** *p* < .01

*** *p* < .001.

After adjustment for socio-demographic variables, PWB is not associated with participation in CBP, while it is independently associated with household income (Table 4).

**Table 4:** Regression models of psychological well-being.

	Model 1		Model 2		Model 3	
	B (SE)	Beta	B (SE)	Beta	B (SE)	Beta
PG vs N-PG	1.17 (1.18)	.06	0.76 (1.13)	.04	-36.3(14.0)	-1.72*
Age, y	-0.05 (0.11)	-.03	0.05 (0.11)	.02	-0.19(0.14)	-.10
Female vs male	-1.27 (1.49)	-.05	-0.31(1.48)	-.01	-0.24(1.46)	-.01
Married vs others	-0.70 (1.47)	-.03	-0.62(1.41)	-.03	-0.80(1.40)	-.04
Education, y	0.24 (0.20)	.08	0.23(0.19)	.07	0.23(0.19)	.08
Log income	8.34 (2.58)	.22**	5.52(2.50)	.15*	5.44(2.47)	.15
Satisfaction with health			2.32(0.67)	.20***	2.31(0.66)	.20***
Doctor visits >6 (yes vs no)			1.26(1.34)	.05	1.36(1.33)	.06
Dependency in IADL (yes vs no)			-2.90(1.21)	-.14*	-2.79(1.20)	-.13*
Cognitive deficit (yes vs no)			-2.83(1.69)	-.09	-3.12(1.68)	-.10
Social network			0.30(0.11)	.16**	0.29(0.11)	.16**
PG x Age					0.52(0.20)	1.76**
R^2^		.08		.18		.20
R^2^ change				.11***		.02*
F		4.12***		5.90***		6.10***

SE–standard error of B; PG–Participant group; IADL–Instrumental activities of daily living.

* *p* < .05

** *p* < .01

*** *p* < .001.

**Fig 1:**
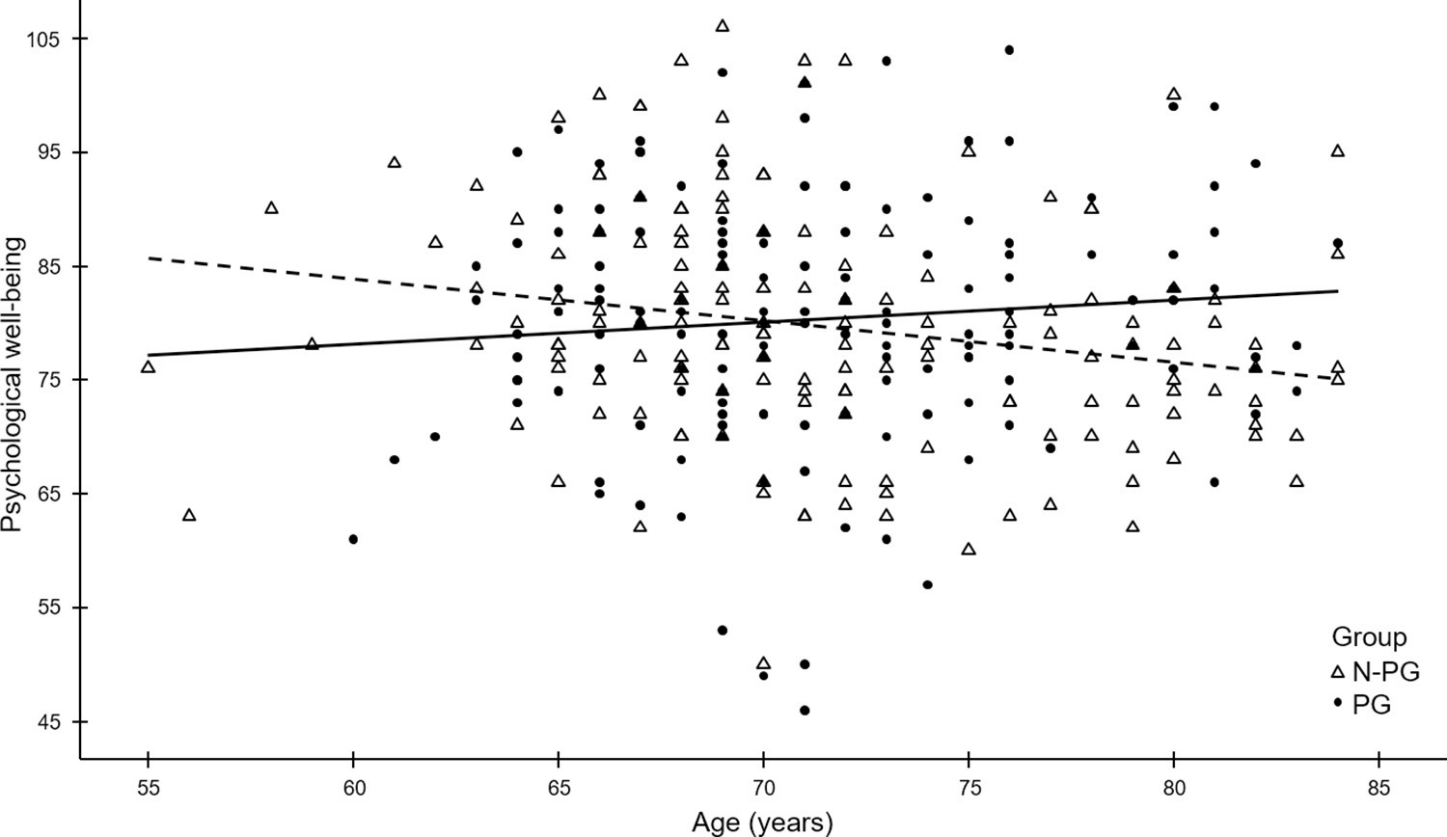
Psychological well-being in CBP participants and non-participants according to age.

As shown in Table 4, adjusting further for health/social variables, PWB is positively associated with satisfaction with health and social network and negatively associated with restrictions in instrumental activities of daily living. After the inclusion of all interactions with socio-demographic variables, there was only a significant interaction with age (S3 Table), so the final model includes this interaction (Table 4). As shown in Fig 1, PWB increases with age in participants, and a contrasting trend was found in non-participants. Using the two age-groups of the matched design, we performed separate analyses for the 55–74 years and 75–84 years old (S4 and S5 Tables). In the youngest, PWB is not associated with participation in CBP, but positively associated with income, satisfaction with health and social network and negatively related to cognitive deficit. In the oldest, PWB is higher in participants, increasing with a growing social network and decreasing in the presence of restrictions in instrumental activities of daily living. Moreover, the only independent predictor of both personal growth and purpose in life in the oldest is participation in CBP.

**Fig 2:**
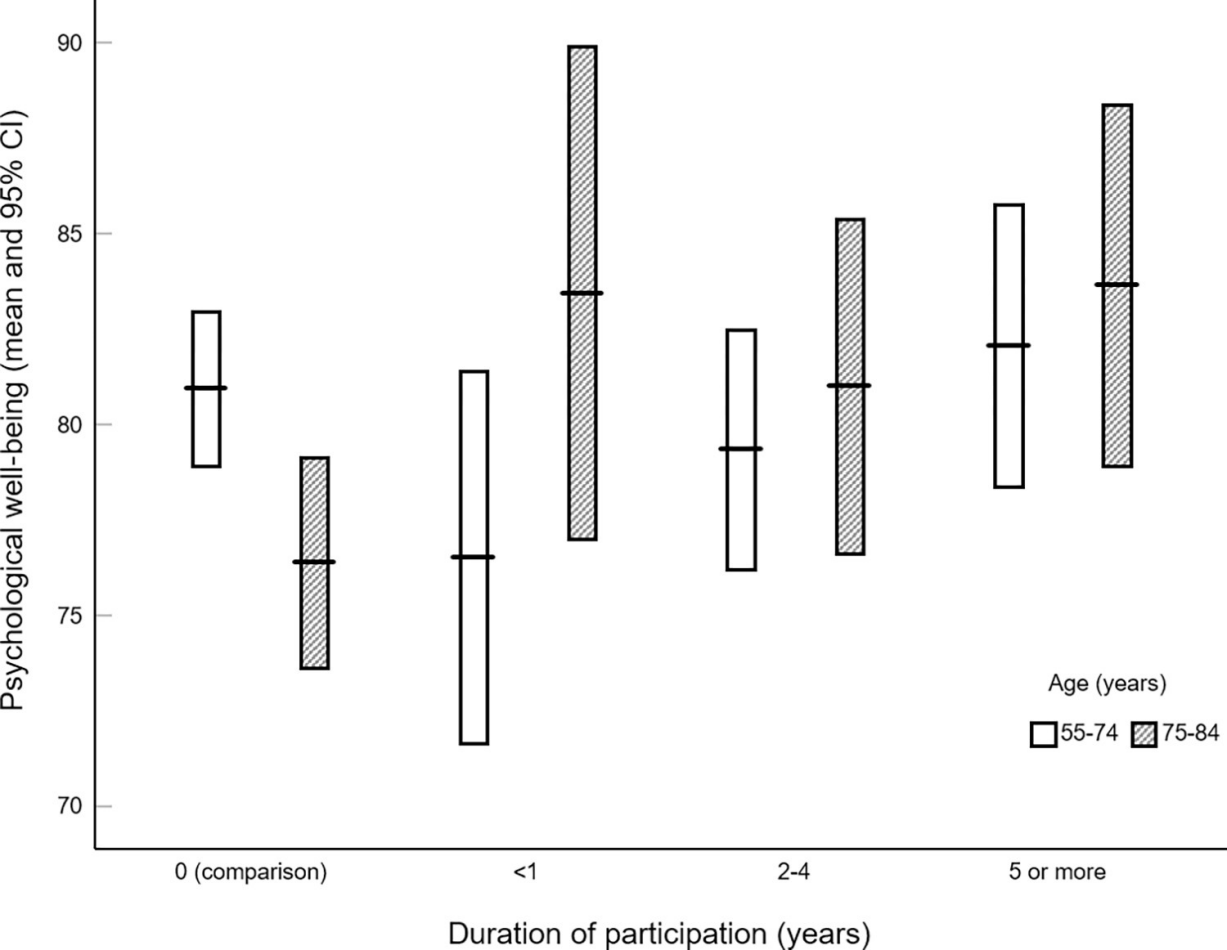
Psychological well-being in different age groups according to the duration of participation in CBP.

Since older participants are more likely to be engaged in CBP for longer periods, to disentangle possible effects of duration of participation and aging on PWB, we used variance analysis to test the effect of duration on PWB in the two age groups above. As shown in Fig 2, in the youngest, there was no significant effect of duration of participation on PWB, *F* (3,212) = 1.67, *p* = .17, while in the oldest PWB increases with duration, *F* (3,84) = 3.82, *p* = .013 as evidenced by a linear contrast, *F* (1,84) = 5.46, *p* = .022.

## Discussion

This study pretends to characterize the population that participates in community-based programs (CBP) and to explore possible benefits in psychological well-being (PWB) in the long run. Overall we can not conclude that participation in CBP contributes to higher psychological well-being levels (H1). Nevertheless, we have shown that the effects of participation in CBP on PWB are inherently linked to the aging process by counteracting the expected decline after 75 years of age (H2). These effects are experienced in personal growth and purpose in life, dimensions previously associated with declines in old age [24,26]. Moreover, we have shown that, after 75 years of age, levels of PWB tend to increase as the length of participation increases.

Staying healthy is the predominant motivation (67%) for being engaged in CBP. So, it is not surprising that most CBP are focused on physical activities offered in all localities, and so most participants (78%) practice physical activity, particularly those engaged in just one activity (60%). Sociocultural and lifelong learning activities are equally offered and attended by approximately 26% of participants. Most participants (57%) occupy themselves at least twice a week, and two-thirds have been engaged in activities for more than one year, with almost all (97%) stating that they attend the activities assiduously. Therefore, participation in CBP appears to be a part of participants’ lives and weekly routines, revealing effective program involvement. These results reflect how the positive view of aging expanded from theory to policy and individual proactivity. In fact, frequency and extension of participation in CBP can be seen as a manifestation of investment in meaningful activities and in social relationships, in line with Rowe and Kahn’s [11] notion of engagement with life. It can also be seen as the type of socially-oriented proactive behavior that favors successful aging, according to Kahana and cols. [13–15]. Lastly, participation in CBP reflects facets of the active aging policy framework promoted by the WHO [5,42].

CBP are mainly addressed to retired people, with low education levels and lower household incomes than non-participants. Why these programs attract such a demographically homogeneous group is a relevant topic for future research, along with exploring the features and effects of engagement with life in individuals with different sociodemographic characteristics.

Regarding health, despite few participants report two or more chronic health conditions, almost half (46%) need assistance in some routine activity of daily living. On average, they see the doctor regularly and more than half affirm attending activities to keep themselves healthy. Overall, the majority is satisfied with their health. At first sight, these results seem to suggest some discrepancies between participants’ objective health status, health behaviors and subjective health. However, a possible explanation is that these individuals are proactively dealing with aging challenges instead of not having challenges at all. As shown by Ryff, Radler and Friedman [28], there are associations between higher PWB and better health, with the former exerting a protective effect over the latter. Santini et al. [43] reach complementary conclusions. These authors show that formal social participation partially protects from chronic disease through its impact on mental health, by enhancing quality of life and reducing depressive symptoms. Considering the present study, perhaps participation in CBP indirectly contributes to health through PWB, particularly for those aged 75 years or older, as will be explored later. On the other hand, maybe CBP participants constitute a group especially committed to adjust to the biological and social aging changes, in the sense conveyed by Caprara and Mendoza-Ruvalcaba [2]. These questions represent interesting avenues for future research.

In both participants and non-participants in CBPs, educational attainment, household income and satisfaction with health contribute to higher levels of PWB, which is in accordance with previous research. Ryff [25] states that those with higher educational levels show higher levels of eudaimonic well-being, but there is similar evidence regarding other well-being outcomes. Former studies found that education is a significant predictor of subjective well-being–life satisfaction and positive affect [44], as well as personal well-being [45]. Silva [46] observed that education, income and subjective health state are significant predictors of hapinness in the Portuguese context. Consequently, these factors should be considered as PWB determinants, particularly to identify potentially vulnerable individuals and groups that might benefit from specific policies and interventions.

Nevertheless, contrasting trends are associated with PWB in the two groups. While in participants PWB builds predominantly upon the social network, which is not so crucial in non-participants, negative aspects of aging, such as age itself and restrictions in daily activities and cognitive deficits, do not carry so much burden for PWB as in non-participants. In fact, previous research shows that age is associated with lower subjective well-being–life satisfaction and positive affect [44]; However, in this study, CBP participants’ well-being seems less susceptible to the detrimental effects of aging. These trends emerge from personal growth and, in particular, from purpose in life since PWB in participants is not associated with age, while in non-participants it tends to decline with aging. Interestingly, previous evidence shows precisely these two dimensions evince decremental age profiles [24,26,47]. Ryff [25,47] proposed that these declining trends in personal growth and purpose in life could reflect the inadequacy of social structures to accommodate longevity. In that sense, CBP might represent the opposite: opportunities for continued growth and development, for the construction of goals and meaning in life.

When modelling the effect of all variables, we may conclude that participating in CBP does not have significantly impact PWB, while income level, satisfaction with health, and social network contribute to higher levels of PWB, and disability in activities of daily negatively affects PWB. However, in the final model, when considering further the joint effect of CBP and all demographic variables, satisfaction with health, social network and functional disability remain significant predictors of PWB. Futhermore, there is a joint effect of CBP and age, translated in the increase in PWB levels with age in participants and an opposite trend in non-participants (see Fig 1). Sub-group analysis by age reveals further that PWB as well as personal growth, and purpose in life before 75 years of age is predominantly linked with the above variables, and not dependent on participation in CBP. On the other hand, after 75 years of age, participation in CBP is the more important factor contributing to higher levels of PWB. Social network and restrictions in instrumental activities of daily living still significantly affect overall PWB, but not personal growth or purpose in life.

This sub-groups analysis corroborates the overall analysis findings, demonstrating that participation gains happen after 75 years of age. Since older participants may have been engaged in CBP for longer, it was essential to analyze the possible effects of participation duration in both groups. In the young, duration of participation does not affect PWB, while in the oldest PWB levels increase with the duration of participation (see Fig 2).

Although regarding a different outcome, previous research [48,49] has highlighted how the association between social activity and quality of life intensifies with age. With age, quality of life increases in very socially engaged individuals, while it decreases in those who are not socially engaged [48], meaning that social engagement becomes more relevant or beneficial as people age. Similarly, our results suggest that engagement in CBP is particularly advantageous for the oldest participants. Perhaps its gains become more prominent as old age challenges accumulate, claiming for more proactive adaptations. Furthermore, participation in CBP might be especially relevant for well-being dimensions known to be more vulnerable in late adulthood since, after 75 years of age, it was the only significant predictor of personal growth and purpose in life. This supports the idea that losses in personal growth and purpose in life during old age reflect a lack of opportunities within social structures to invest in meaningful roles and activities, as Ryff [25,46] proposed. It also corroborates the notion that human beings are plastic organisms and that human development entails both gains and losses at any given point in the life cycle [2,8].

### Limitations and strengths

Concerning study limitations, we can not point out to beneficial effects of specific activities (physical, sociocultural or lifelong learning) on PWB. Such analysis was not possible due to the reduced number of participants that do not attend physical activities. Still, since more than 70% are engaged in physical activities, and the primary motive for participation is staying healthy, beneficial effects are necessarily linked to these activities. That is, if we were to implement similar programs, we would first propose their inclusion.

Also, individuals in the study might naturally perform activities similar to the ones implemented in CBP in a non-structured way. However, apart from asking participants if they took part in any other activities, this study specifically aimed to compare individuals that participate in CBP with those who do not attend such programs, considering that these initiatives have specific qualities, namely social aspects and a formal organization.

Interpretation of the findings should take into account sociodemographic characteristics of the matched samples that are not significantly different, thus including, in general, persons with low educational levels and low incomes, but this defines the target population of these programs. Moreover, participants in these CBP are residents of relatively small towns and we may not generalise results to other distinct environments.

Additionally, causality in the associations between CBP participation and PWB can not be assumed since the data are cross-sectional, and at the same time, reverse causality is a possibility. That is, better PWB might contribute to involvement in CBP, whereas lower PWB might negatively affect participation. It is reasonable to think that there are reciprocal effects between activity participation and well-being. Nevertheless, the association between participation in CBP and PWB in the older age group was significant even when accounting for sociodemographic, health and social characteristics. It was the only significant predictor of both personal growth and purpose in life.

It should also be mentioned that used measures rely extensively on self-reports which could have led to under or overestimating some variables. Even though these measures did not involve long recall periods and, in some cases, namely outcomes, the focus was precisely on subjective perspective. Finally, the study focuses on comparisons between CBP participants and non-participants. More detailed measurement scales of the quantity and quality of participation in CBP could allow for a more nuanced analysis.

Despite these limitations, this study has the advantage of comparing paired groups that did not significantly differ in socio-demographic characteristics, lending support to our findings. Also, it includes data from three Portuguese territories, which adds diversity in terms of the opportunities and constraints that shape the aging process. Furthermore, the present research shows how CBP may impact older adults’ PWB using data from natural settings and real municipal programs implemented to promote successful aging. Hence, it explores actual applications of policy measures, addressing a relevant research gap with significant implications for policy and practice.

### Future research, policy and practice

The fact that participants in CBP have a very homogeneous socio-demographic profile raises questions about how and why different population groups structure around social engagement activities. So, there is a need for further inquiry concerning these socio-demographic clusters and, simultaneously, the social engagement patterns of other groups, namely men, single people, those with higher educational attainment and income, and those with more vulnerable health, functional ability, and social network.

As already mentioned, subsequent studies might benefit from an analysis that goes beyond comparisons between participants and non-participants in CBP to include more detailed aspects of activity participation, such as intensity, involvement level, motivation level, and perceived benefits or drawbacks.

Longitudinal designs allow testing of the reversed causality hypothesis mentioned earlier. Moreover, it is important to monitor the development of CBP participants and non-participants by collecting data at more than one point in time. This study can also help clarify if participation in CBP indirectly contributes to health via PWB or if these programmes act as buffers in the face of biopsychosocial ageing-related changes.

Lastly, it seems essential that this study extends to older ages. On the one hand, our results suggest that CBP are particularly relevant for those aged 75 and older. On the other, the fourth age (85 years or older) is marked by a higher incidence of functional disability and other losses that may interfere with PWB, which is particular important for designing public policies, community interventions and services that respond to aging challenges.

Involvement in CBP can provide opportunities to enhance individual resources protective of PWB in late adulthood. Such opportunities seem to be especially valuable for those aged 75 years and older, and for well-being dimensions known to decline in late life. In fact, findings from the present work hint at relevant policy and practice implications. Since age, dependency and cognitive deficits were associated with lower PWB in non-participants only, it is possible that CBP exerts a protective effect against aging losses that are undesirable per se but also threaten well-being.This points to the possibility of counteracting age deleterious effects by combining societal and individual efforts to build up older adults’ reserve capacity to respond to aging challenges.

## Conclusion

In conclusion, PWB is linked to healthy aging, defined by WHO [7, p. 9] as “The process of developing and maintaining the functional ability that enables well-being in older age”. We may add that while PWB is not associated with functional disability and cognitive deficits in participants, in non-participants, PWB declines as these obstacles increase. Since groups do not differ according to these characteristics, we may conclude that these “functional abilities/disabilities” do not affect PWB in participants contrasting to non-participants, contributing to its maintenance.

Despite the encouraging results of this study, given the cross-sectional nature of data, we can not guarantee whether cumulative effects of CBP can result in gains of PWB. Only longitudinal studies involving follow-up of participants can answer this and other questions, namely possible buffer effects of CBP in the PWB of persons with low IADL performance and/or cognitive deficits.

## Supporting information

S1 Table Sample of participants by gender and age-group in each locality. (PDF)

S2 Table Activities performed during last year by participants (PG) and non-participants (N-PG). (PDF)

S3 Table Regression models for testing effects of PG on relation between socio-demographic variables and psychological well-being. (PDF)

S4 Table Regression models of psychological well-being for the 55–74 age group (*N* = 216). (PDF)

S5 Table Regression models of psychological well-being for the 75–84 age group (*N* = 88). (PDF)
