# Supplementary material for: Engagement with life and psychological well-being in late adulthood: Findings from community-based programs in Portugal
Source: PLoS One. 2023 May 19;18(5):e0286115. doi: 10.1371/journal.pone.0286115 (PMC10198493; doi:10.1371/journal.pone.0286115)
Supplement: S1 Table — (PDF) [file pone.0286115.s001.pdf]

S1 Table

Supplemental Table 1.

Sample of participants by gender and age-group in each locality

Gender /Age-group (years)	All	Municipality A	Municipality B	Municipality C
Women	114 (75.0)	37 (74.0)	42 (80.8)	35 (70.0)
55-64	10 (8.8)	4 (10.8)	2 (4.8)	4 (11.4)
65-74	76 (66.7)	26 (70.3)	26 (61.9)	24 (68.6)
75-84	28 (24.6)	7 (18.9)	14 (33.3)	7 (20.0)
Men	38 (25.0)	13 (26.0)	10 (19.2)	15 (30.0)
55-64	2 (5.3)	1 (7.7)	0 (0.0)	1 (6.7)
65-74	20 (52.6)	6 (46.2)	6 (60.0)	8 (53.3)
75-84	16 (42.1)	6 (42.2)	4 (40.0)	6 (40.0)
