# Supplementary material for: Engagement with life and psychological well-being in late adulthood: Findings from community-based programs in Portugal
Source: PLoS One. 2023 May 19;18(5):e0286115. doi: 10.1371/journal.pone.0286115 (PMC10198493; doi:10.1371/journal.pone.0286115)
Supplement: S2 Table — (PDF) [file pone.0286115.s002.pdf]

S2 Table

Supplemental Table 2.

Activities performed during last year by participants (PG) and non-participants (N-PG)

Activities	All (N = 304)	PG (n = 152)	N-PG (n = 152)
Going to restaurants/coffee shops/cultural or sports events	228 (75.0)	110 (72.4)	118 (77.6)
Travelling with other people	204 (67.1)	108 (71.1)	96 (63.2)
Visiting/inviting friends, relatives or acquaintances	270 (88.8)	134 (88.2)	136 (89.5)
Helping others (trasportation, shopping, housekeeping)	259 (85.2)	128 (84.2)	131 (86.2)
Participating in religious activities	258 (84.9)	135 (88.8)	123 (80.9)
Member of recreational/cultural/educational associations*	115 (37.8)	68 (44.7)	47 (30.9)
Member of volunteer association (hospital/charity/prison)	46 (15.1)	24 (15.8)	22 (14.5)
Walking/jogging/biking/swimming/dancing/fishing	39 (12.8)	22 (14.5)	17 (11.2)
Yardwork/housework/gardening	35 (11.5)	19 (12.5)	16 (10.5)

*Chi-square test = 6.2, $p = 0.013$
