# Supplementary material for: Engagement with life and psychological well-being in late adulthood: Findings from community-based programs in Portugal
Source: PLoS One. 2023 May 19;18(5):e0286115. doi: 10.1371/journal.pone.0286115 (PMC10198493; doi:10.1371/journal.pone.0286115)
Supplement: S3 Table — (PDF) [file pone.0286115.s003.pdf]

S3 Table

Supplemental Table 3.

Regression models for testing effects of PG on relation between socio-demographic variables and Psychological well-being

	Model 2		Model 3	
	Beta	T value	Beta	T value
Age, y	.02	.39	-.11	1.41
Female	-.01	.23	-.07	.86
Married	-.03	.45	-.01	.16
Education, y	.08	1.19	.08	.87
Log income	.14	2.16*	.11	1.27
Satisfaction with health	.20	3.53***	.20	3.44***
Doctor visits >6	.05	.94	.05	.98
Dependency in IADL	-.14	2.41*	-.13	2.20*
Cognitive deficit	-.09	1.69	-.11	1.96
Social network	.16	2.80**	.16	2.75**
PG			-2.02	2.47*
PG x Age			1.95	2.68**
PG x Female			.15	1.15
PG x Married			-.05	.36
PG x Education			.01	.05
PG x Log income			.06	.64
R^2		.18		.21
R^2 change		.11***		.03
F		6.45***		4.67***

PG – Participants group; IADL – Instrumental activities of daily living
Value for │t│; *p < .05 **p < .01 ***p < .001
