# Supplementary material for: Engagement with life and psychological well-being in late adulthood: Findings from community-based programs in Portugal
Source: PLoS One. 2023 May 19;18(5):e0286115. doi: 10.1371/journal.pone.0286115 (PMC10198493; doi:10.1371/journal.pone.0286115)
Supplement: S4 Table — (PDF) [file pone.0286115.s004.pdf]

Supplemental Table 4.

Regression models of psychological well-being for the 55-74 age group (*N* = 216)

	PWB		Personal growth		Purpose in life	
	Beta	T value	Beta	T value	Beta	T value
PG	-.06	.89	.02	.24	-.00	.06
Age, y	-.06	.89	-.02	.25	-.02	.33
Female	-.01	.16	-.01	.16	-.01	.17
Married	-.12	1.64	-.23	3.23**	-.01	.15
Log income	.22	2.96**	.23	3.18**	.17	2.30*
Satisfaction with health	.19	2.81**	.25	3.76***	.19	2.66**
Dependency in IADL	-.08	1.17	-.02	.21	-.03	.41
Cognitive deficit	-.16	2.48*	-.01	.17	-.10	1.53
Social network	.17	2.61**	.14	2.20*	.14	2.09*
R^2		.20		.22		.13
F		5.63***		6.27***		3.39***

PG – Paticipants group; IADL – Instrumental activities of daily living

*p < .05 **p < .01 ***p < .001
