# Supplementary material for: Engagement with life and psychological well-being in late adulthood: Findings from community-based programs in Portugal
Source: PLoS One. 2023 May 19;18(5):e0286115. doi: 10.1371/journal.pone.0286115 (PMC10198493; doi:10.1371/journal.pone.0286115)
Supplement: S5 Table — (PDF) [file pone.0286115.s005.pdf]

Suplemental Table 5

Regression models of psychological well-being for the 75-84 age group (*N* = 88)

	PWB		Personal growth		Purpose in life	
	Beta	T value	Beta	T value	Beta	T value
PG	.30	3.06**	.22	2.04*	.37	3.69***
Age, y	.13	1.18	.06	.53	.21	1.96
Female	.00	.02	.01	.08	.00	.00
Married	.14	1.19	.06	.45	.14	1.22
Log income	.12	1.09	.16	1.29	.16	1.40
Satisfaction with health	.19	1.93	-.02	.15	.05	.53
Dependency in IADL	-.20	2.02*	-.03	.29	-.16	1.53
Cognitive deficit	.03	.28	-.01	.10	-.08	.77
Social network	.21	2.01*	.19	1.67	.11	1.04
R^2		.29		.14		.27
F		3.59**		1.44		3.25**

PG – Participants group; IADL – Instrumental activities of daily living
**p* < .05 ***p* < .01 ****p* < .001
